# Spatial Dynamics of the Communities and the Role of Major Countries in the International Rare Earths Trade: A Complex Network Analysis

**DOI:** 10.1371/journal.pone.0154575

**Published:** 2016-05-03

**Authors:** Xibo Wang, Jianping Ge, Wendong Wei, Hanshi Li, Chen Wu, Ge Zhu

**Affiliations:** 1 The State Key Laboratory of Management and Control for Complex Systems, Institute of Automation, Chinese Academy of Sciences, Beijing, 100190, China; 2 School of Humanities and Economic Management, China University of Geosciences (Beijing), Beijing, 100083, China; 3 Key Laboratory of Carrying Capacity Assessment for Resource and Environment, Ministry of Land and Resources, Beijing, 100083, China; 4 College of Government, Peking University, Beijing, 100871, China; 5 School of Economics, Peking University, Beijing, 100871, China; 6 Structural Geology Group, China University of Geosciences (Beijing), Beijing, 100083, China; 7 Beijing Key Laboratory of Water Resources and Environment Engineering, China University of Geosciences (Beijing), Beijing, 100083, China; Shanxi University, CHINA

## Abstract

Rare earths (RE) are critical materials in many high-technology products. Due to the uneven distribution and important functions for industrial development, most countries import RE from a handful of suppliers that are rich in RE, such as China. However, because of the rapid growth of RE exploitation and pollution of the mining and production process, some of the main suppliers have gradually tended to reduce the RE production and exports. Especially in the last decade, international RE trade has been changing in the trade community and trade volume. Based on complex network theory, we built an unweighted and weighted network to explore the evolution of the communities and identify the role of the major countries in the RE trade. The results show that an international RE trade network was dispersed and unstable because of the existence of five to nine trade communities in the unweighted network and four to eight trade communities in the weighted network in the past 13 years. Moreover, trade groups formed due to the great influence of geopolitical relations. China was often associated with the South America and African countries in the same trade group. In addition, Japan, China, the United States, and Germany had the largest impacts on international RE trade from 2002 to 2014. Last, some policy suggestions were highlighted according to the results.

## Introduction

Rare earths (RE) are performing important functions in our everyday life, with their widespread use in a range of products [1] and modern industries, including clean energy, environmental protection, aero-space, and electronic information industries. Although RE are not rare in a geological sense, the distribution of RE reserves is not even. Currently, there are 130 million tons of RE proven reserves globally [2]. Nearly half of these reserves are located in China (42.31%), with Brazil accounting for 16.92% and Australia accounting for 2.46%. Sizeable deposits are also found in Brazil, India, Australia, and Canada [3].

Because of the global RE uneven distribution and important functions for national development, most of countries obtain REs by import from the international market. From 1940s to 1980s, the United States was the leading supplier in the RE international market. The majority of RE exported from the United States to the rest of the world was produced from the Mountain Pass mine in California, which is one of the best minable deposits in the world [4, 5]. However, in the 1990s, China’s RE production increased sharply; as a result, China exported a large amount of RE to other countries, thereby lowering the RE prices and weakening the share of RE supply of the USA in the international supply market [6]. Moreover, given the increasing ecological costs as a result of a halt in chemical processing in 1998 caused by a series of wastewater leaks, the Mountain Pass mine ended operations in 2002 [7]. Subsequently, China gradually became the world’s largest RE producer; in 2014 China had 86.36% share of the global production, and China was also the world’s largest RE exporter, with the export quotas of 31,000 tons [2]. Due to environmental protection and resources conservation, the Chinese government started to regulate RE exports through export duties and export quotas beginning in 2006, resulting in a decline in exports [8]. Though the World Trade Organization upheld a ruling in favor of the claims of the United States, the European Union, and Japan that China violated trade rules with respect to the unfair imposition of export restrictions on RE [2], China will continue to inhibit illegal production and exports via implementation of domestic regulation on resources exploitation and environmental pollution in the RE production [8]. In contrast to China’s export decreasing, global demand of RE was expected to increase at a steady annual growth rate in excess of 5% from 2014 to 2020 [2]. The volume and value of global trade in RE has been continuously changing. Moreover, the number of RE trade participating countries reached its maximum in 2012 and has since gradually declined [9]. Therefore, the world’s RE trade will continue to evolve; in particular, suppliers will be more diversified to meet the increasing demand.

Current studies on international RE trade focus on China’s RE supply and demand and policies on exports. Mancheri evaluated China’s position in international trade in terms of both volume and value and proposed some policy implications on state support and financial incentives for domestic mining and processing as well as vertical integration in the supply chain for the countries other than China [9]. Chen determined that the international RE shortage will reach 18,000 to 50,000 tons if China’s export quotas are set between 32,000 tons and 35,000 tons [10]. In the case of China's restrictions on RE exports, Castor and Hedrick indicated that the Mountain Pass RE Mine in the United States may be reopened, with its RE output depending on China's export restrictions, price increase, and the growth of domestic demand and other factors [11]. Based on China's RE Development Plan (2009–2015) and media reports, Wübbeke analyzed China's RE export policy and the development trend of China's RE terminal industry and indicated that by 2015, there will be no new RE mine projects and predicted that by 2015, China's RE output will be maintained between 130,000 tons and 150,000 tons and the level of exports will be less than 35,000 tons [3]. He et al. introduced a multi-attribute decision-making methodology, which is then used to select the optimal RE trading partner [6]. The results showed that Japan would be the optimal trading partner for Chinese RE. Wang et al. forecasted China’s RE production by mine type [12]. Based on the findings, policy recommendations were proposed for China and other countries. The findings also indicated that countries without RE resources must prepare to face the risk of price rises, especially for ion-absorbed RE in the international market. Han et al. analyzed adjustments in China's RE regulation policies and the effects on the RE market supply by using an astatic game-theoretic model and a dynamic game-theoretic model [8]. The results show that when abolishing RE export restrictions, China's RE production and export will increase, whereas RE production, domestic supply and price in foreign country will decrease. Moreover, if the Chinese government enhances resource taxes and levies an environmental tax, then the production, domestic supply, and exports of RE will decrease in China, whereas the opposite effects will exist in other countries.

The existing literature has provided solid empirical investigations and represents a good reference for understanding the international trade of RE. However, the previous research studies have mainly focused on the growing RE supply crisis, soaring Chinese RE export prices, and China’s RE trade policies. The majority of the studies considered RE international trade issues based on experiential judgment of the supply capacity of RE states, rather than on quantitative modeling. Some of the literature reports analyzed RE international trade using a quantitative model; however, these studies are limited to the analysis RE international trade between the major countries.

In fact, the real international trade is a complex system with numerous countries and complicated relations. The development of complex network theory has offered an effective method for the research of international trade, with numerous researchers successfully using complex network theory to study international trade, especially in the aspect of uneven distribution resources and energy. Currently, there are increasing studies on international trade under the network framework [13, 14, 15, 16, 17, 18, 19, 20, 21, 22, 23, 24, 25, 26, 27, 28]. Among the studies, complex network analysis is applied to natural resources, such as natural gas trade [24] and oil trade [25, 27, 28]. Moreover, complex network is also adopted in the study of coupled biological and chemical systems, neural networks, and social interacting species which are the systems composed by a large number of highly interconnected dynamical units [29]. In particular, the complex network theory is widely used to understand dynmics in spatially structured ecological networks [30]. Studies [31, 32, 33, 34, 35, 36] could be highlighted in the discussions on the roles of spatial diffusion in networks. However, the dynamics and its influencing factors of international RE trade have not attracted sufficient attention and discussion.

We will leverage the complex network applicable for analysis of the international trade of uneven distribution resources and energy to analyze the international trade of RE. In this paper, the unweighted and weighted network model of the international RE trade were established to explore the evolution of communities. Our models contain data for 15 years from 2002 to 2014. We use an algorithm based on the concept of modularity to detect the RE community in the network of global RE trade. We depict communities by colored world maps; analyze the evolution of communities by community scale and modularity, use the Normalized Mutual Information index (NMI) to measure the stability of the communities, and analyze the evolution of degree value between the international RE trade network to identify the major countries’ position. Thus, the paper makes three contributions. First, our study includes most countries in the international RE trade, and thus, providing a comprehensive and systemic analysis on the distribution and internal relations between trade groups as well as the changes in the countries within the trade group, which differs the current studies on the RE trade between inter-individual countries. Second, a normalized mutual information is introduced to our study to describe the stability of the partition in the international RE trade, which helps us to grasp the trend of international RE trade. Third, this is the first study to portray the role of countries participating in the international RE trade based on the degree and strength values of node.

## Methodology and Data

### Methodology

Countries are represented as nodes here. Trade relations between countries are represented as edges, whose directions denote the RE trade flows and whose weights denote the quantities of RE trade. A complex network is formed based on the annual data of international RE trade.

#### Communities and Globalization

Communities are clusters of countries in our study. In most cases, the relations between countries in the same community are stronger than the relations between countries in different communities [28]. We apply the detecting-algorithm developed by Blondel et al. [37] to separate the network into communities. Modularity as an index is used to measure the quality of the partition obtained by the above detecting-algorithm. The modularity of a partition, which has a scalar value between -1 and 1, evaluates the density of links inside communities compared to links between communities. The higher the value of modularity is, the better the partition will be [28]. The modularity of a partition (Q) can be defined as below:

$$\text{Q}=\frac{1}{2m}\Sigma_{i.j}\left[w_{i.j}-\frac{A_{i}A_{j}}{2m}\right]\delta \left(c_{i},\;c_{j}\right)$$ (1)

where *w*_*i*.*j*_ is the weight of the edge between nodes i and j. In the unweighted network model, when a trade relation exists between the two countries, the value will be 1; otherwise, it will be 0. *A*_*i*_ = Σ_*j*_ *w*_*i*.*j*_ is the sum of the weights of the edges attached to node i. *c*_*i*_ is the community where node i is allocated. *δ*(*c*_*i*_, *c*_*j*_) is 1 if *c*_*i*_ = *c*_*j*_ and is 0 otherwise. $\text{m}=\frac{1}{2}\sum_{i,j}w_{i,j}$.

There are two phases repeating in an iterative way in the algorithm to measure the trade communities. First, each node i is taken as a community, so the number of communities is same as that of nodes. In addition, for node i, when i is placed into its neighboring community of j, the algorithm measures the gain of modularity ΔQ. Taking into consideration every neighboring community of node i, if the gain is positive, then i will join the one with the maximum ΔQ; otherwise, it will remain in its original community. When the process is repeatedly and sequentially performed for all nodes and no further improvement can be achieved, the first phase of the algorithm ends. The gain of modularity ΔQ is calculated using Eq 2 [37].

$$\Delta \text{Q}=\left[\frac{{\sum^{\text{​}}}^{\text{​}}C_{in}+A_{i,in}}{2m}-{\left(\frac{{\sum^{\text{​}}}^{\text{​}}tot+A_{i}}{2m}\right)}^{2}\right]-\left[\frac{{\sum^{\text{​}}}^{\text{​}}in}{2m}-{\left(\frac{{\sum^{\text{​}}}^{\text{​}}tot}{2m}\right)}^{2}-{\left(\frac{A_{i}}{2m}\right)}^{2}\right]$$ (2)

In (Eq 2), Σ *C*_*in*_ is the sum of the weights of all the links inside community C, Σ *tot* is the sum of the weights of the links incident to all nodes in community C, including the links inside community C and the links between the node in community C and the node out of community C, *A*_*i*_ is the sum of the weights of the links incident to i, *A*_*i*,*in*_ is the sum of the weights of the links from i to all nodes in C, and m is the sum of weights of all links in the network.

In the algorithm’s second phase, a new network will be built up, and its nodes will be the communities found during the first phase. The weights of the links between the new nodes are decided by the sum of the weights of the links between nodes in the corresponding two communities. Edges between nodes of the same community will have self-loops in the new network.

When the second phase is completed, the first phase of the algorithm will be applied once again to the resulting network, and then this process will be iterated. The iteration of these two phases continues until no more changes occur; the result is the maximum modularity.

By merging nodes and clusters from the original network, the hierarchy of the network can be determined, and the uncovered hierarchical structure may be observed with the desired resolution. Thus, this algorithm can circumvent the resolution limit problem. In addition, this algorithm is also fast and easy to implement because the gains in modularity are easy to compute.

#### Stability

To study the stability of the partition that we obtained, a measure to quantify the statistical information shared between two distributions is introduced here, named the Normalized Mutual Information, or NMI [38]. When the value of the NMI increases, the partition will be more stable. The algorithm below evaluates the MNI between two neighboring years:

$$NMI_{(y^{\left(t\right)},y^{\left(t+1\right)}}==\frac{{\sum^{\text{​}}}_{h=1}^{k^{\left(t\right)}}{\sum^{\text{​}}}_{l=1}^{k^{\left(t+1\right)}}n_{h,l}log\left(\frac{n\times n_{h,l}}{n_{h}^{\left(t\right)}n_{l}^{\left(t+1\right)}}\right)}{\sqrt{({\sum^{\text{​}}}_{h=1}^{k\left(t\right)}n_{h}^{\left(t\right)}log\frac{n_{h}^{\left(t\right)}}{n})({\sum^{\text{​}}}_{l=1}^{k\left(t+1\right)}n_{l}^{\left(t+1\right)}log\frac{n_{l}^{\left(t+1\right)}}{n}}}$$ (3)

where *y*^(*t*)^ denotes the year t, $n_{h}^{\left(t\right)}$ denotes the number of nodes in cluster h in year t, $n_{l}^{\left(t+1\right)}$ is the number of nodes in cluster l in year t+1, and *n*_*h*,*l*_ represents the number of nodes both in cluster h in year t and in cluster l in year t+1. In the whole network, n is the number of nodes.

#### Trading role

In the traditional analysis of complex network, identifying whether the nodes play a key role is one of the main topics. One of the centrality measures is the degree of nodes, which are evaluated by the number of their trade partners. The country with a higher degree of node will have more influence in the structure of the network [39]. The degree of a node i in the multilayer network is measured as below:

$$K_{i}=\overset{3}{\underset{\alpha }{\sum^{\text{​}}}}K_{i}^{\alpha }=\overset{3}{\underset{\alpha =1}{\sum^{\text{​}}}}\overset{n}{\underset{j=1}{\sum^{\text{​}}}}\alpha_{ij}^{\alpha }$$ (4)

${\text{K}}_{\text{i}}^{\alpha }$ is the degree of the node i in layer α.

The difference in the trade intensity in the weighted network will cause each edge to have an inconsistent impact on the trade network. For weighted layers, node strength of i can be defined as (Eq 5). The strength of country i is the sum of the weights of the edges attached to the country. For a given country, if the node strength becomes higher, then the country will have more trade influence.

$$W_{i}\;=\;\sum_{\alpha =1}^{3}W_{i}^{\alpha }\;=\;\sum_{\alpha =1}^{3}\sum_{j=1}^{n}W_{ij}^{\alpha }$$ (5)

### Data

We built a network model using the data on international RE trade downloaded from UN Comtrade, which contains all import flows among the trade participating countries in the world. The name of the commodity is “Rare-earth metals, scandium and yttrium” (the HS code is 280530). Export data cannot fully reveal the truth in RE trade because RE are often smuggled, although import data can reduce the errors to some extent. In addition, RE can be divided into many types, including mixed RE, bastnasite, ion-absorbed RE, etc. Ion-absorbed RE, which are orders-of-magnitude higher in price than mixed RE because of the lower reserves, plays an extremely important part in the RE trade. The share of ion-absorbed RE will be minimized if a network is built with trade volume. As a result, we selected the annual import value data of RE trade for countries from 2002 to 2014 on the basis of the two concerns mentioned above.

## Results and Discussion

### Communities

Based on the data of RE trade values, we applied the algorithm in section 2.1.1 to both unweighted networks and weighted networks of the model, and Table 1 shows the number of communities in the two types of networks. The unweighted network model describes the international RE trade only by relationships. The weighted network model describes the international RE trade by quantities to show the actual relationship between the countries. The difference between the partition of the unweighted and weighted network reflects the difference between the RE relationships and the actual relationships.

**Table 1:** Number of communities.

Year	2002	2003	2004	2005	2006	2007	2008	2009	2010	2011	2012	2013	2014
Unweighted	7	6	5	7	6	9	8	8	9	7	6	7	7
Weighted	5	8	6	8	5	5	8	7	7	5	5	5	4

**Fig 1:**
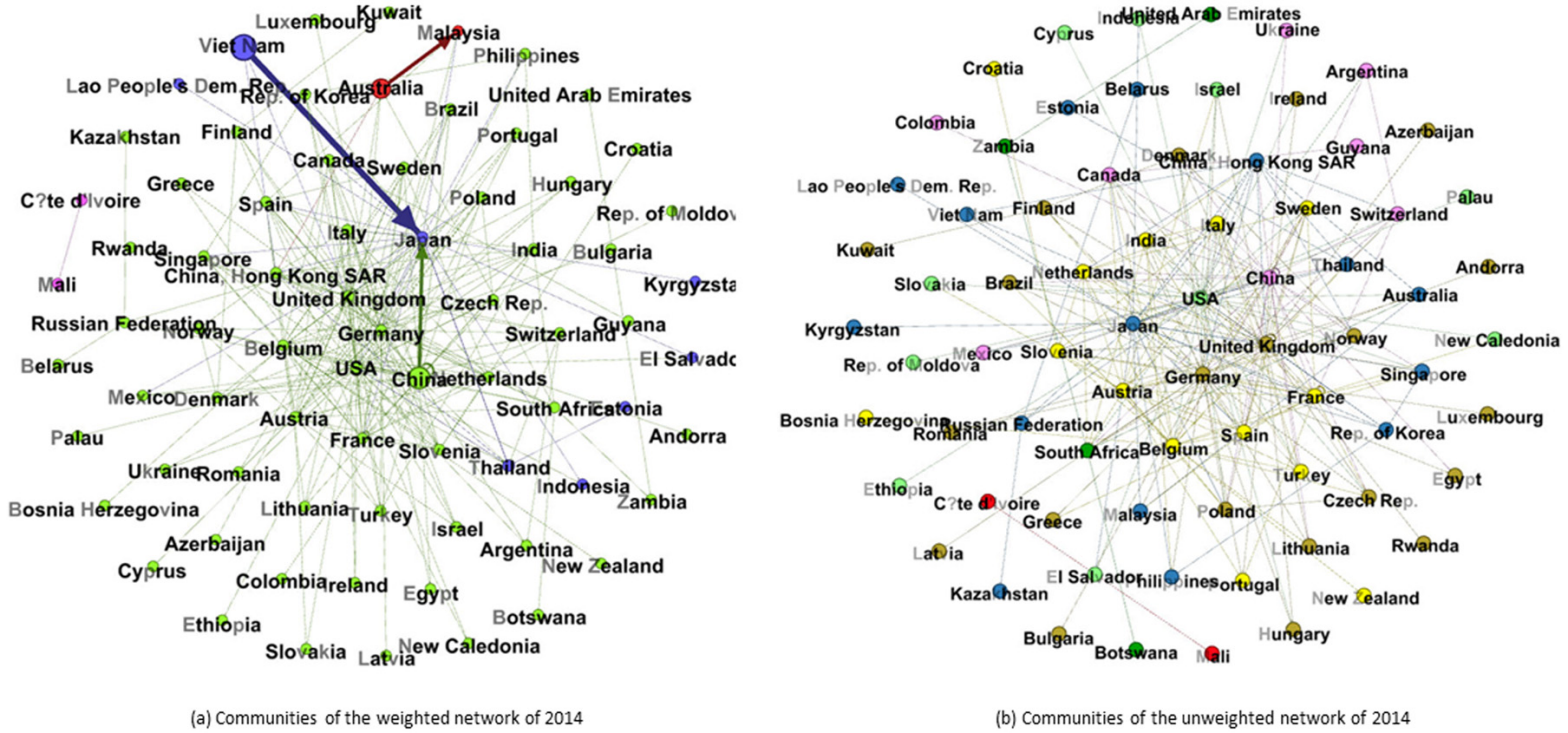
Communities of the weighted and unweighted network of 2014. (A) The nodes in the figure indicate the countries. (B) The links represent the trade relationships between two countries. (C) The thicknesses of links show the value of the trade between two countries. (D) The arrow represents the direction of the trade flow. (E) The nodes in the same color are in the same communities.

The real RE trade is composed of numerous countries and trade links; therefore, there are thousands of trading relationships. We took the weighted and unweighted network in 2014 as an example to show the trading countries, relationships and communities in Fig 1. The nodes in same color means they are in the same communities.

Table 1 shows that the number of trade communities in unweighted network has changed between six and nine, and that in weighted network varied between four and eight in the past 13 years.

The changing number of trade communities presents a bell-like curve. In an unweighted network, the number of trade communities changed from seven in 2002 to nine in 2010 and gradually merged into seven in 2014; as for the weighted network, it changed from five in 2002 to eight in 2008 and gradually merged into four in 2014. Coincidentally, during the years from 2002 to 2014, when the number fluctuated, countries other than China had relatively stable policies on RE. It can be concluded that the fluctuation of RE trade communities is related to the policy in China, the world’s major supplier of RE.

### Evolution of the communities

#### Globalization

**Fig 2:**
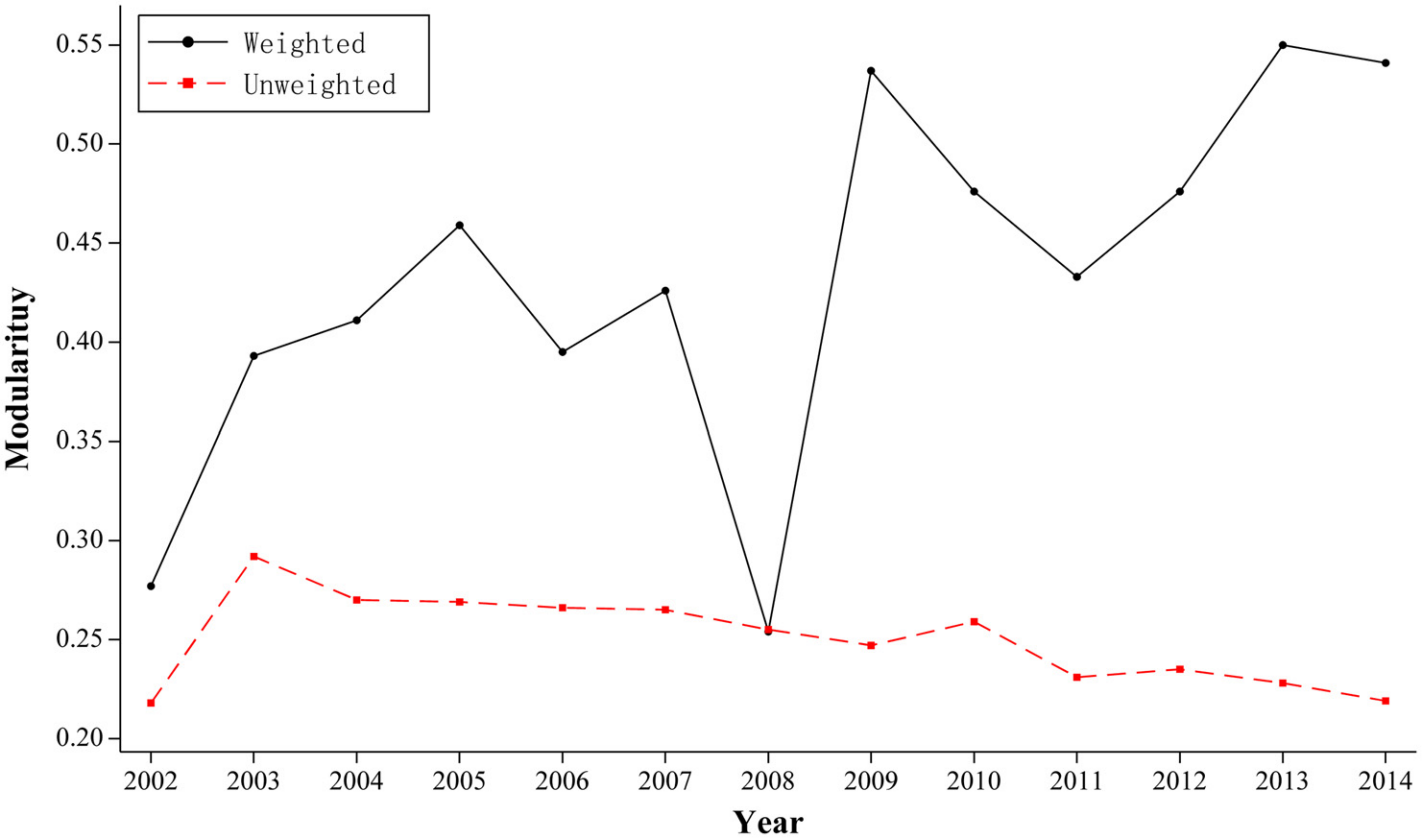
Evolution of modularity. (A) The black line shows the value of modularity in the weighted network. (B) The red line indicates the value of modularity in the unweighted network.

Modularity measures the quality of the partition of the communities. A larger value of modularity represents a better partition of a complex network. We recorded the values of modularity while detecting communities in the unweighted and weighted networks in Fig 2. From Fig 2, we can see that the communities of the weighted network are much more segregated than the unweighted network.

In the unweighted network, the value of modularity descends gradually in the period from 2002 to 2014. This trend indicates that during the 13 years, the relationship between countries in the international RE trade became closer and the divisions among the communities became vaguer.

In the weighted network, the modularity value curve exhibited an enormous increase in the period 2002–2014. This finding indicates that, considering trade amounts, the actual relationship between countries is not as close as their trade relationship shows.

#### Evolution map of the communities

**Fig 3:**
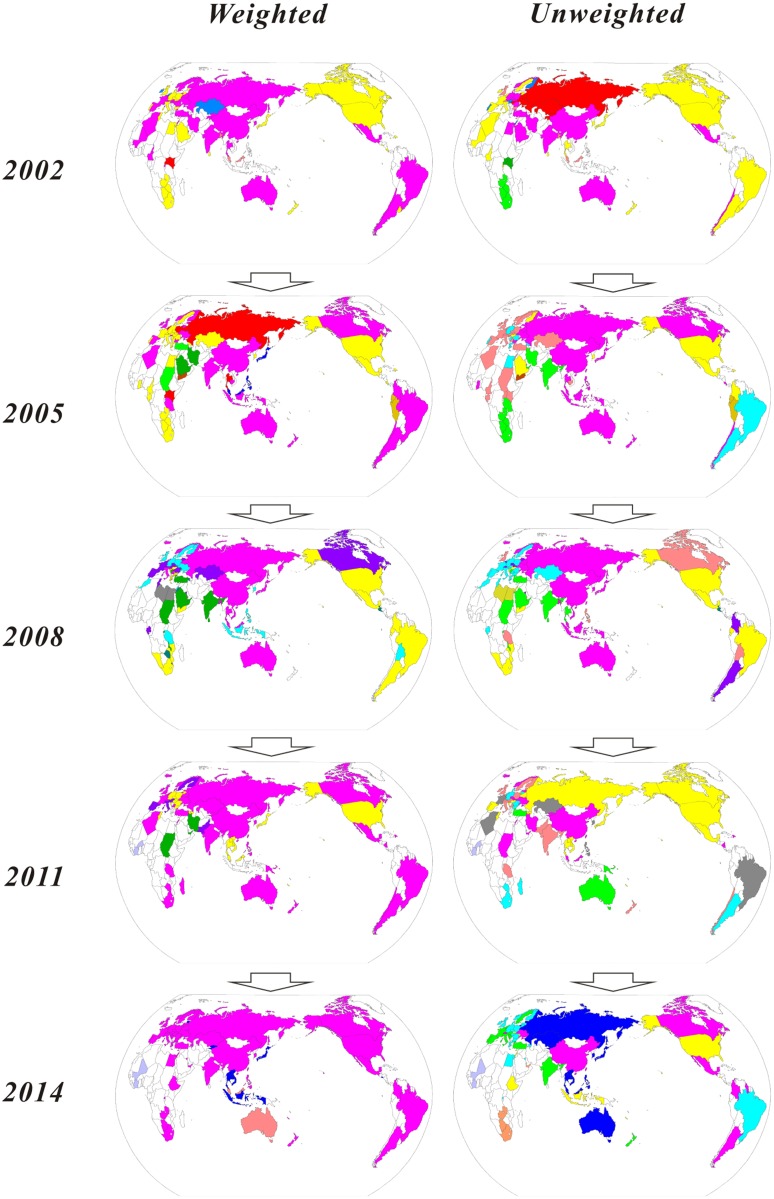
World maps of communities’ evolution. (A) The maps show the international RE trade communities’ evolution from 2002–2014. (B) The countries in the same color are in the same communities. (C) The weighted maps on the left side represent the communities division based on the trade value or weight. (D) The unweighted maps on the right side indicates the communities division without consideration of trade value or weight. Note: The authors performed Fig 3 based on the Fig 4 of [25] using CorelDRAW software with their new statistical data.

To show the international evolution of RE communities in a much clearer way, we mark the same community in the same color in a world map. A country without a mark means it did not participate in the global RE trade that year. Fig 3 shows a map of international evolution of RE communities in five years. The map shows that the formation of trade community is largely affected by regional aspects.

In the map of the unweighted network, with the consideration of only trade relations and not trade volume, trade countries tend to unite geographically closer countries to be a trade community. As for the trend of the unweighted network, there are fewer and fewer trade communities from the previous structure of four main trade countries, namely China, Europe, the United States, and Japan, to the former three gradually merging into one trade community in 2014, indicating closer trade relations among the RE trade countries.

In the map of weighted network, considering trade volume, countries other than China still tend to associate with geographically closer countries to form a trade community, whereas China tends to cooperate with geographically farther ones, e.g., countries in Africa, Latin America, and Western Europe. This behavior occurs because RE are considered as strategic resources, resulting in a close tie between the RE trade community and international geopolitical relations in China.

#### Stability of the trade network

**Fig 4:**
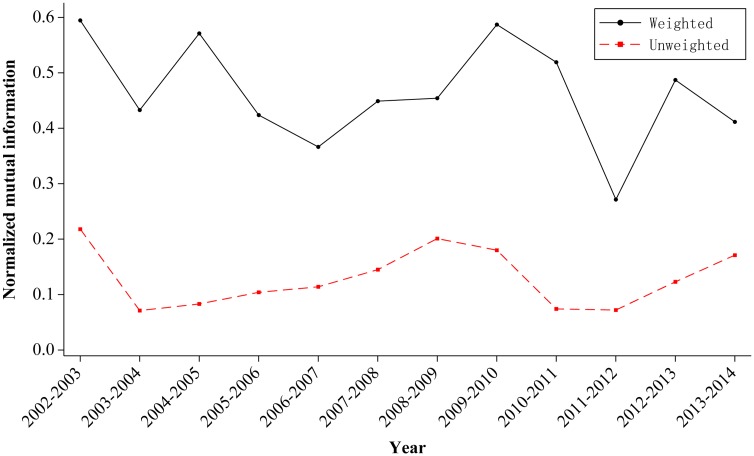
Stability of the network. (A) The black line shows the NMI in the weighted network. (B) The red line indicates the NMI in the unweighted network.

We calculated the NMI in the unweighted and weighted networks of the 13 years; the result is shown in Fig 4. The curves of the weighted and unweighted networks exhibit similar trends of increase or decrease.

First, regarding the overall evolution tendency, the dramatic fluctuation shows the weak stability of the RE trade. The partition of the weighted network is more stable than that of the unweighted network, i.e., the actual relationship in amount of RE trade between countries is more stable than their relationship. Three reasons lead to the weak stability. First, some countries do not participate in global RE trade annually. Second, the safekeeping of RE in RE resource-rich countries has resulted in the condition of lower supply compared to the demand during the past 13 years; thus, countries without resources have been constantly seeking such resources among the trade communities. Third, RE are important resources with uneven distribution and yield, and their trade relations are built on the basis of whether a partner country has export resources. If not, then the trade relations will easily be broken.

**Fig 5:**
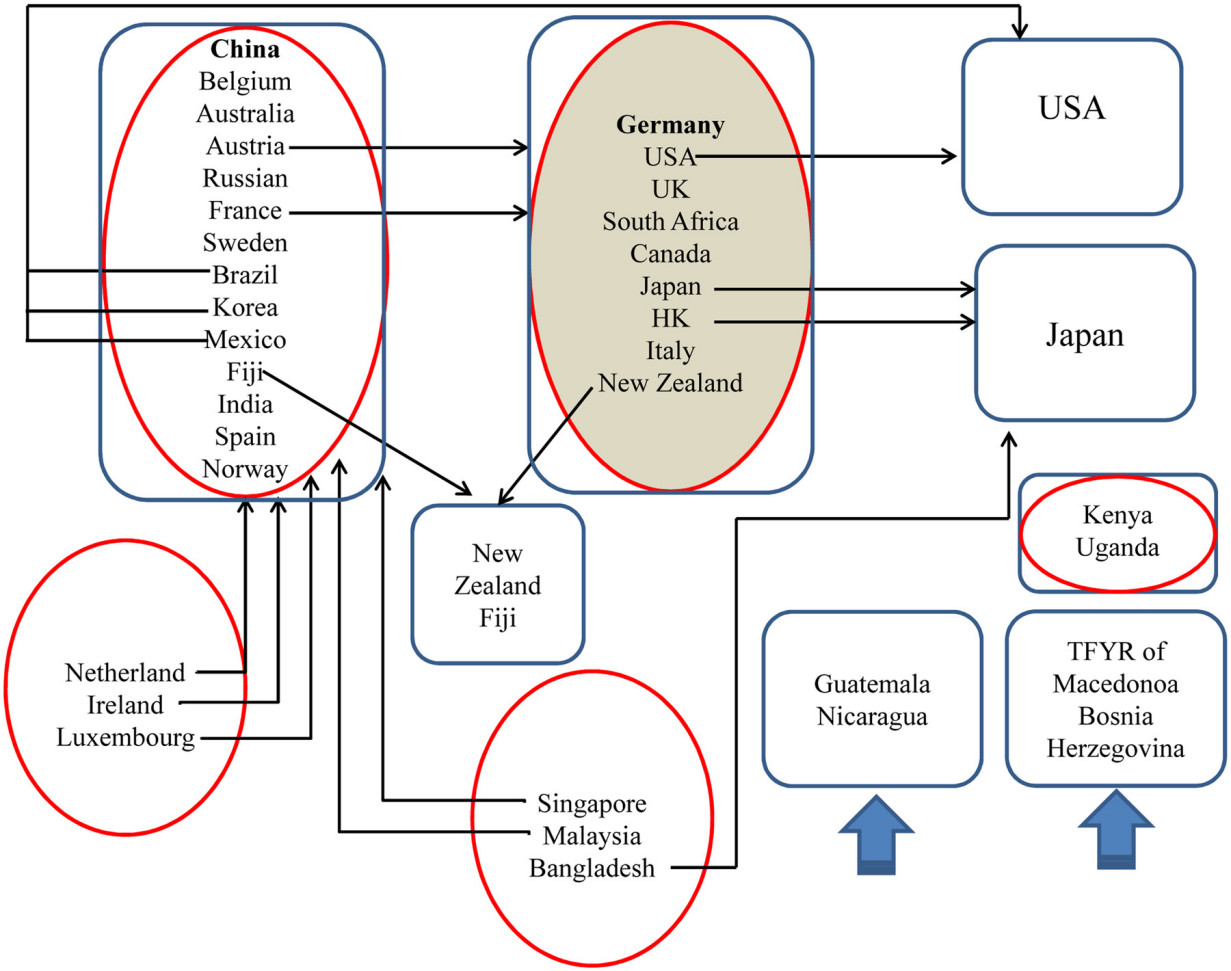
Migration of major countries in 2002–2003. (A) The red circle indicates the communities in 2002. (B) The blue circle represents the communities in 2003. (C) The arrow shows the movement direction of the countries between communities in the period of 2002–2003. (D) The communites above blue thick arrow represent the communities that are new formed and did not participate the international RE trade before.

**Fig 6:**
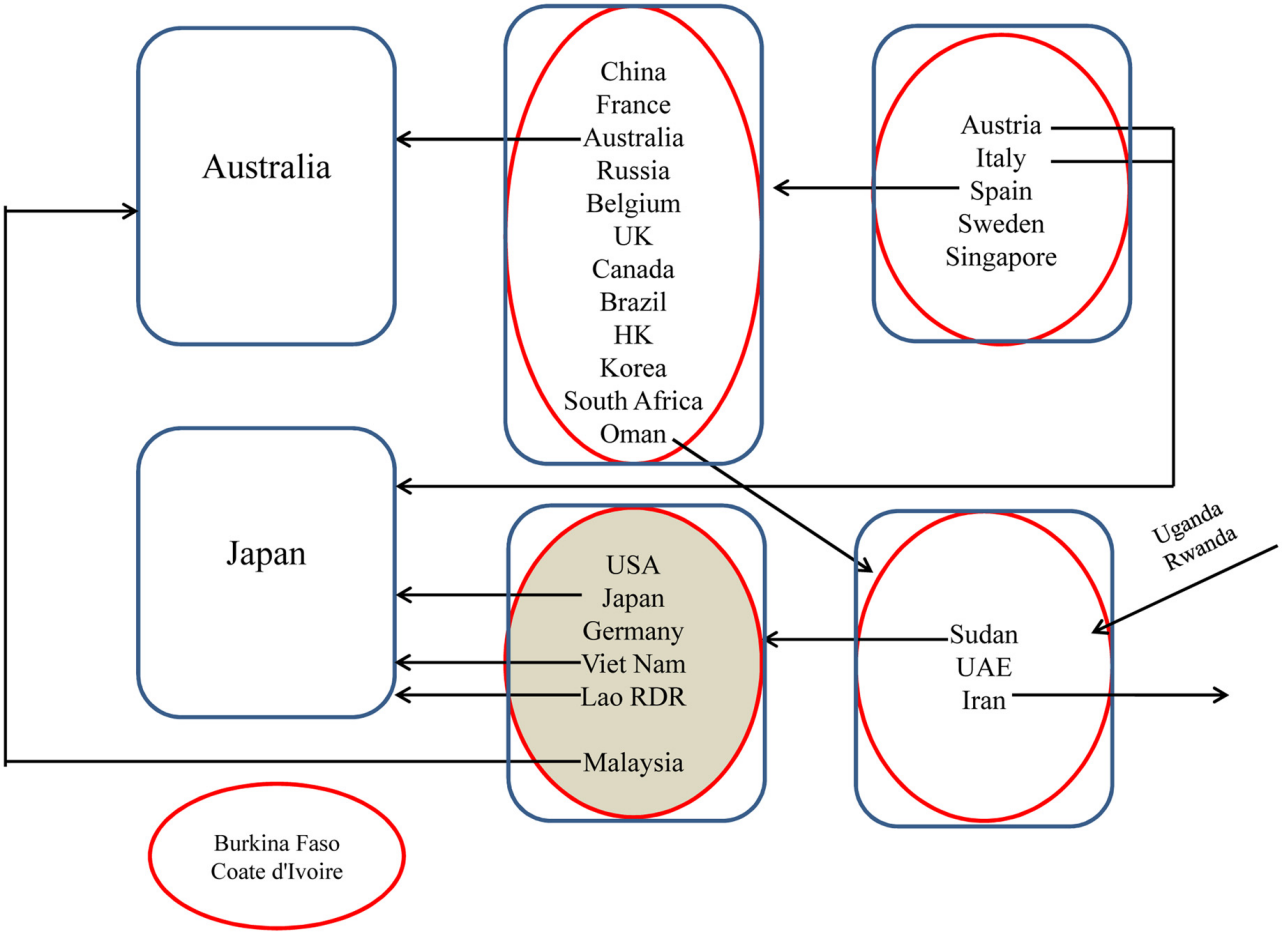
Migration of major countries in 2011–2012. (A) The red circle indicates the communities in 2011. (B) The blue circle represents the communities in 2012. (C) The arrow shows the movement direction of the countries between communities in the period of 2011–2012.

Second, the curve of the weighted network reached its peak point in 2002–2003 and its lowest point in 2012–2013. Through comparison, the two points have something in common. As Figs 5 and 6 show, during the past 13 years from 2002 to 2014, there has been only two years, 2002 and 2011, when the United States, Japan, and Germany were in the same community. It is unclear why the number reached the peak point after 2002 in the point of 2002–2003 and then decreased to the lowest point after 2011 in the point of 2011–2012. We will analyze the two periods of time as follows.

In the figures above, a red circle represents communities in 2002 and 2011, and a blue circle represents those in 2003 and 2012.

(1) The point of 2002–2003. The curve of the weighted one reached its peak in 2002–2003, i.e., global trade network has remained the most stable at that time during the past 15 years. There are two main reasons. First, the number of RE trade countries basically remains unchanged. Comparing the trade-participating countries in 2002 and 2003, we find 60 out of 70 are the same. Second, RE export figures in these two years are quite similar to each other. The following are values from 2002 (2003): global RE export value of 65.5 million (77.8 million) USD (the United States dollar); export figures of 11,200 (13, 900) tons; unit export value of 5,849 (5593) USD per ton.

(2) The point of 2011–2012. The curve of the weighted network decreased to its lowest point in 2011–2012, i.e., the global trade network was the most unstable at that time during the past 15 years. There are two main reasons for this observation. First, the number of RE trade countries increased dramatically from 80 in 2011 to 104 in 2012. Second, RE export figures in these two years have changed the most in the past 13 years. The following are values from 2011 (2012): global RE export value of 800.7 million (344.3 million) USD; export figures of 80,000 (73,000) tons; unit export value of 10,008 (4,715) USD per ton.

From the analysis and comparison above, we determine the reasons of the instability of the trade network: the change of the trade countries and the unit export value. It can be concluded that the policy of increasingly rigorous export quotas on RE in China, the world’s largest exporter, resulted in the rapid increase of the global unit export value to 100 million USD per ton in 2011. As a result, the United States, Japan, and Germany, who were in the same trade community in 2011, eventually joined together and prosecuted China in the WTO for its RE export quotas policy in the year of 2012.

### Evolution of the positions of major countries

RE trade countries have different positions in the international network. The real position of countries in the RE trade system can be reflected through the countries’ position in the network. As shown in Eqs (4) and (5), the position importance of the country in the network rely on the number of its trading partners and the contribution ratio to the economy of its partners. The larger the country’s degree is, the more influence it has in the structure of the network. In this section, we will rank the countries by calculating the weighted degree value, the weighted in degree value, and the weighted out degree value to show their influence on international trade, both import and export.

First, calculate the three values mentioned above of the trade countries in 2014, and then rank them according to the result. Next, for countries listed in the top 50, rank their three values mentioned above from 2002 to 2013, respectively.

Second, assign the ranking index to the top 50 countries: the first is assigned 100, the second is assigned 98, and so on. A larger index indicates that the country plays a more important role and has a stronger influence in international RE trade.

Third, calculate the average of the assignment from 2002 to 2014 and then analyze the countries with an average greater than 70 as well as the RE resource countries.

#### Overall situation analysis

**Fig 7:**
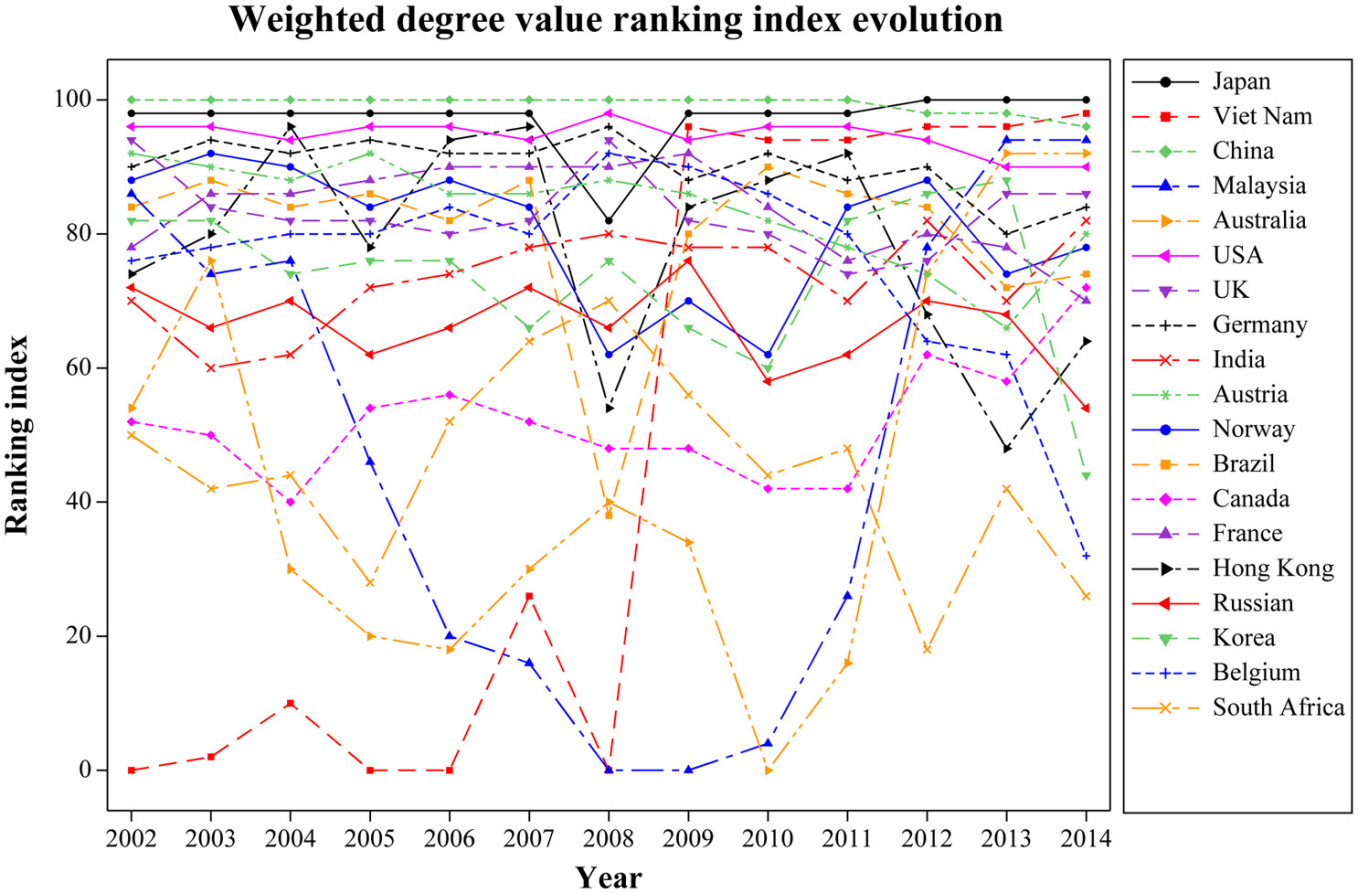
Weighted degree value evolution. (A) The lines in different colors indicate the weighted degree value ranking index evolution of different countries.

According to the ranking index of weighted degree value evolution in international trade of years from 2002 to 2014 (Fig 7), it can be easily seen that countries with the average above 90, including China, Japan, the United States, and Germany, have the strongest influence; non-resource countries and regions, e.g., Austria, UK, France, Norway, Hong Kong, Korea, and Belgium, as well as the resource country of Brazil, with the average between 75 and 85, have some influence; finally, resource countries of Australia, Vietnam, Malaysia, South Africa, Canada, and Russia, with the average below 70, have weaker influence. Among them, Australia, Malaysia, and Vietnam show dramatic growth in the ranking only in this year. In particular, Vietnam, who went through a soaring increase in 2009, finally surpassed China and became the world’s second most influential country in 2014.

**Fig 8:**
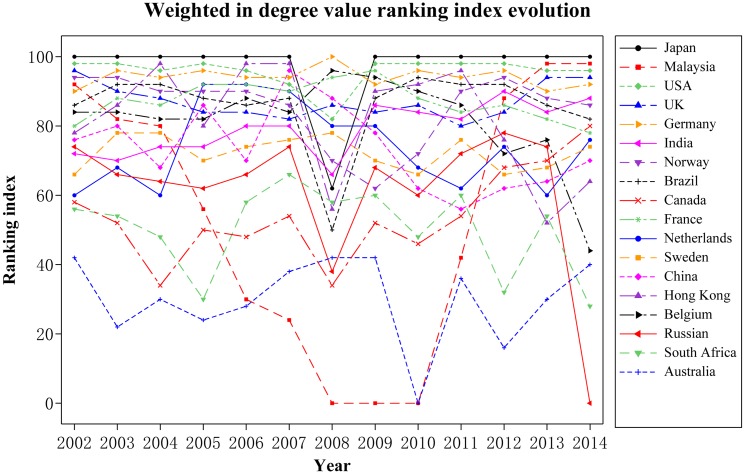
Weighted in degree value evolution. (A) The lines in different colors indicate the weighted in degree value ranking index evolution of different countries.

Regarding RE import trade shown in Fig 8, Japan, the United States, Germany, UK, France, Norway, Brazil, Hong Kong, and Belgium have their average above 80. All of them are non-resource countries, except the United States and Brazil. While most resource countries including India, China, Malaysia, Canada, South Africa, and Australia, as well as non-resource countries, including Holland and Sweden have their average below 80.

As the leader of RE trade countries, Japan plays an important part in international import trade. Its average influence of years from 2002 to 2014 is more than that of resource countries, including Brazil, Canada, Russia, and South Africa. Regarding the RE import trade, non-resource countries, including Japan, UK, Germany, Norway, and France, have a stronger influence. The figure above shows that the ranking of Japan, Malaysia, the United States, India, Brazil, Canada, and Hong Kong largely decreased in 2008 because of the impact of the economic crisis on the RE demand as well as its terminal industries.

**Fig 9:**
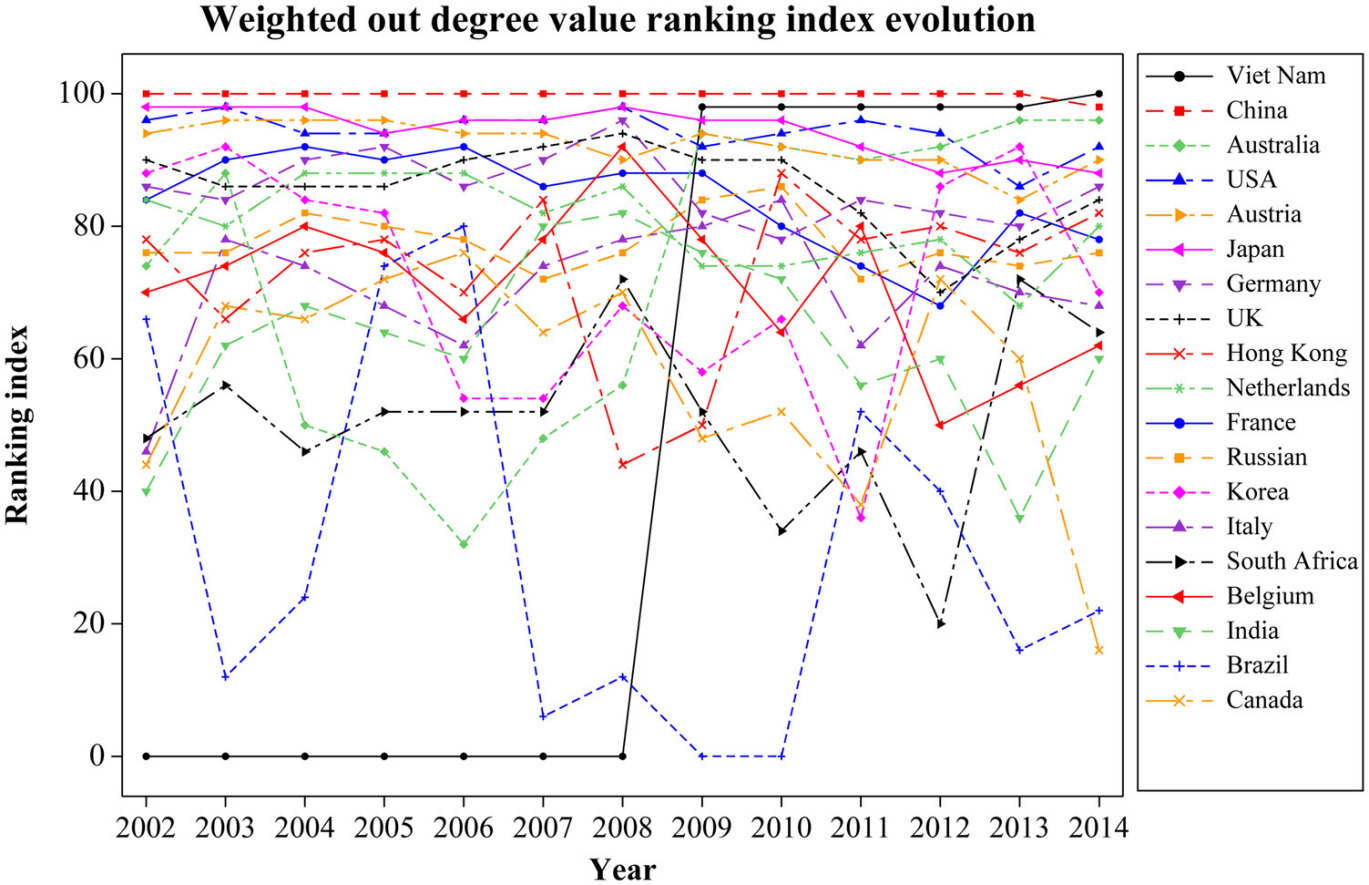
Weighted out degree value evolution. (A) The lines in different colors indicate the weighted out degree value ranking index evolution of different countries.

Regarding RE export trade shown in Fig 9, the countries of China, the United States, Japan, Austria, UK, Germany, France, and Holland have their average ranking indices above 80. RE resource countries, including Australia, India, Canada, South Africa, Vietnam, and Brazil, have their averages below 70. Through comparison, we can see that most RE resource countries, except China, the United States, and Russia, have a weaker influence on export trade. Note that the ranking index of Vietnam rose from 0 in 2008 to 98 in the following year, and it finally surpassed China ranking the first on the list of international RE export trade, which helped Vietnam become the second most important country regarding the global RE trade. With China’s policy on RE export quotas as the background, we believe that the rising of the ranking index is because Vietnam, who enjoys close geopolitical relations with the United States and Japan, started to export RE to the two countries.

**Fig 10:**
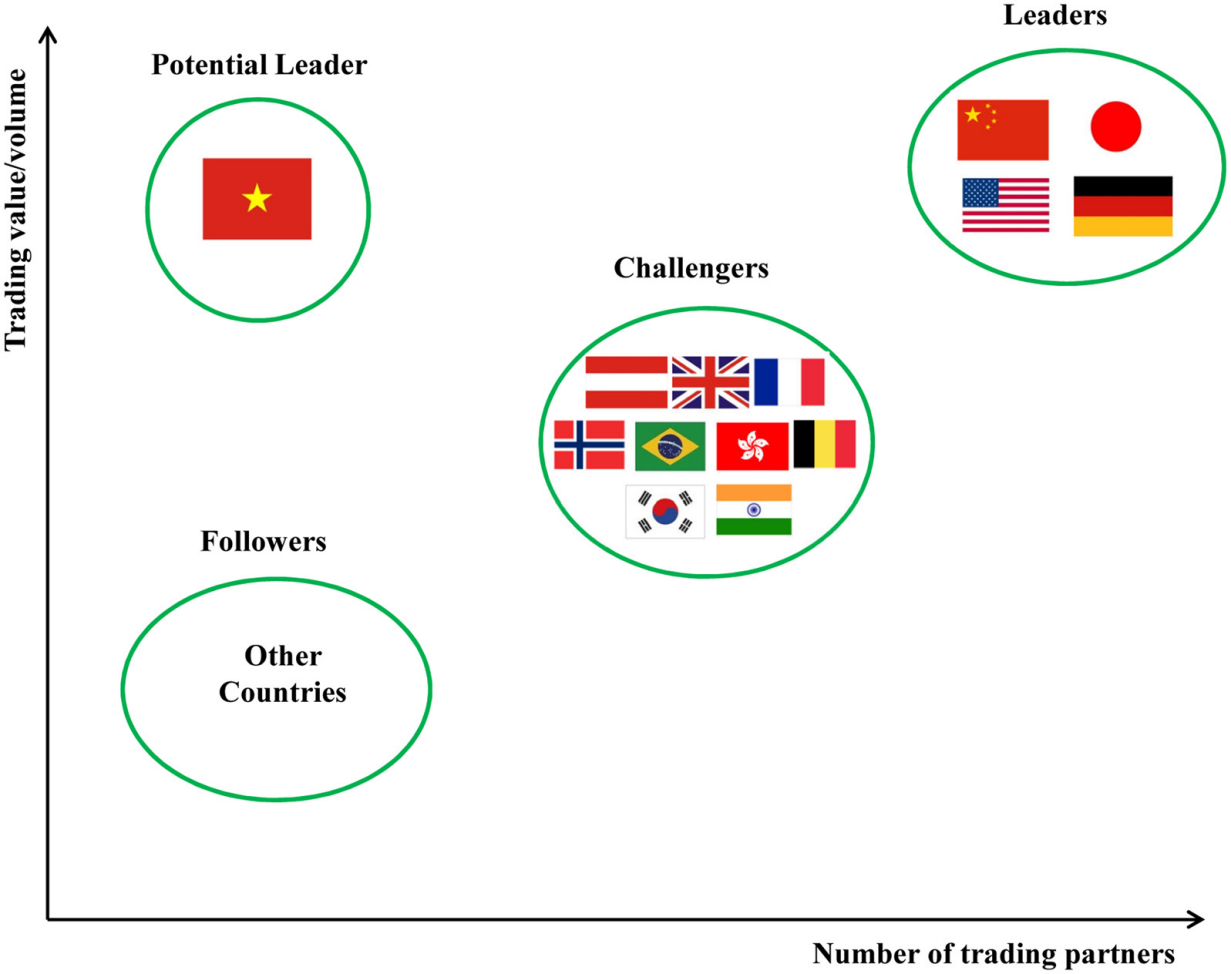
Division of the RE trade group. (A) Group 1 in a green circle defined as leaders includes China, Japan, the United States, and Germany. (B) Group 2 in a green circle defined as challengers includes Austria, UK, France, Norway, Brazil, Hong Kong, Belgium, Korea, and India. (C) Group 3 in a green circle defined as potential leader includes Vietnam. (D)Group 4 in a green circle defined as followers includes other countries.

According to Table 2, in which we have calculated average ranking index of the major RE trade countries in the world from 2002 to 2014, the countries can be divided into four groups shown in Fig 10. Group 1 includes China, Japan, the United States, and Germany. We define these four countries, of each which with an average above 90, as the “Leaders”, i.e., they play decisive roles in international RE trade and leading the development of the global RE industry. Group 2 includes Austria, UK, France, Norway, Brazil, Hong Kong, Belgium, Korea, and India. We define these nine countries and regions, with each of their average between 70 and 90, as “Challengers”, i.e., they are important trade-participating countries in international RE trade and play important roles in their local market. Group 3 includes Vietnam. We define it as a “Potential leader”. The ranking index of Vietnam, with only 40.8 as its average, rose from 0 in 2008 to 96 in the following year, and it finally surpassed China to rank the first on the list of international RE export trade. We regard Vietnam as a potential leader because of its low average, but Vietnam is very likely to be one of the most important countries in international RE trade in the future. Group 4 includes the countries for which the average of each is below 70. These are trade-participating countries in international RE trade who play certain roles in promoting the market.

**Table 2:** Averages of the ranking indices.

	Total	Import	Export
China	99.47	76.40	99.87
Japan	97.33	97.47	94.93
USA	94.80	96.00	94.53
Germany	90.67	94.27	86.13
Austria	84.53	-	92.00
UK	84.40	87.73	86.53
France	83.60	86.53	83.47
Norway	80.80	85.33	-
Brazil	79.47	85.33	28.53
Hong Kong	79.20	82.00	73.87
Belgium	75.60	80.80	72.40
Korea	72.80	-	71.73
India	71.20	77.33	61.33
Russian	66.27	61.60	77.73
Canada	53.73	56.00	54.93
Malaysia	52.00	57.47	-
South Africa	43.73	49.20	49.87
Australia	41.33	30.00	69.47
Viet Nam	40.80	-	39.33

#### Leader and potential leaders analysis

(1) China: China is currently the largest producer and exporter of RE in the world. Comparing the data of import trade with the export trade in Table 3, it can be seen that the latter is larger than the former, i.e., China plays the role of an exporter in the international RE trade. Before 2012, China was the most important country in the international RE trade. However, China’s position began to fall in 2012, with China being surpassed by Japan and Vietnam in 2012 and 2014, respectively. From 2008, both the trend of the RE import and export figures and the number of trade partners of China declined. In 2012, these quantities decreased to their lowest points in history, resulting in China’s influence on global RE trade to decrease to third-ranked in the world.

**Table 3:** China’s RE trade situation.

	Ranking index			Trade Value		Trade Volume		Number of trading partners		
	Total	Import	Export	Import	Export	Import	Export	Total	Import	Export
2002	100	76	100	773184	48538396	29942	8981007	28	7	23
2003	100	80	100	668386	59208287	60843	11621788	30	8	23
2004	100	68	100	401900	83309540	38369	12240702	28	5	28
2005	100	86	100	1291881	112071476	41987	12986661	27	6	27
2006	100	70	100	422168	185160395	33947	13677323	27	7	27
2007	100	96	100	17452705	307186607	332257	12458157	27	7	27
2008	100	88	100	2354246	166128382	199630	6961371	21	6	20
2009	100	78	100	666015	72689735	30793	5345099	26	6	26
2010	100	62	100	714434	178674799	1180	6262289	21	5	20
2011	100	56	100	855365	603719694	2268	3240692	15	6	14
2012	98	62	100	1587420	187364238	23367	2756811	15	4	15
2013	98	64	100	1373783	111546069	12322	3106858	19	5	18
2014	96	70	98	1003200	77649914	5731	3738816	15	4	15

The reason for the decline is that Chinese government introduced various policies to manage the RE trade since 2006. One type of trade regulation policy was the tariff adjustment policy. The Chinese government began to collect tariffs for RE imports of 10% and increased it up to 25% in 2012. Another type of trade regulation policy was the policy of export quotas. The Chinese government implemented the policy of reducing export quotas in 2006, and the export quotas decreased 39% in 2008 and the same percentage in 2010, resulting in the continuous decline of export figures and the reduction in the number of trade partners. Finally, there were more policies, including the policy of controlling and forbidding of primary RE products, and the policy of the qualification management of RE export enterprises. The Chinese government has forbidden the export of most primary RE products since 2008. Meanwhile, the number of RE export enterprises declined gradually from 2008 to 2011.

(2) Japan: Japan is a non-resource trade country in the world. Since 2011, the RE trade volume of Japan has begun to surpass that of China. It can be seen from the comparison of its export figures and import figures that Japan is more active in import than export in a large degree, leading to the continuous rise of its position in international RE trade.

From the changing trend of the ranking index, there are two years to which we should pay more attention, as shown in Table 4. One is the year of 2008, when Japan’s total ranking index decreased to 82 because of the global financial crisis; in addition, the import ranking index of Japan dropped to 62, the lowest in history. Another one is the year of 2012, when Japan’s global RE trade value and number of trade partners all surpassed those of China, resulting in the ranking index of Japan surpassing that of China to become the most important country of RE trade in the world.

**Table 4:** Japan’s RE trade situation.

	Ranking index			Trade Value		Trade Volume		Number of trading partners		
	Total	Import	Export	Import	Export	Import	Export	Total	Import	Export
2002	98	100	98	31614799	1290983	4985145	176616	13	10	8
2003	98	100	98	36754158	1644443	6119122	174916	9	6	7
2004	98	100	98	51903034	1198859	6379212	277204	12	5	10
2005	98	100	94	84018728	2377977	8387335	591809	12	9	7
2006	98	100	96	148688122	5222934	9460742	1610459	15	8	9
2007	98	100	96	240723889	3516628	9320137	614768	12	5	10
2008	82	62	98	165948179	2782730	6306728	290437	13	5	11
2009	98	100	96	77951006	1514526	4772969	23157	9	5	8
2010	98	100	96	170222205	4195062	5487037	28291	11	6	7
2011	98	100	92	714949741	3815897	5210651	9512	12	8	8
2012	100	100	88	400528168	15728465	4792881	198363	17	11	10
2013	100	100	90	201854223	13917408	5193798	274840	17	11	9
2014	100	100	88	226347914	8834030	6479437	132040	13	10	8

(3) USA: The United States, an important country with RE resources, enjoys more than 10% of the total reserves in the world. It can be seen from the comparison of the export figures and import figures of the United States that the influence of export and import of the United States are almost the same, except in 2008, when the global financial crisis caused the decline of the import influence of the United States. A comparison shows that the United States has different features from China and Japan regarding RE trade. Unlike China, whose export figures are far larger than its import figures, and Japan, whose situation is exactly the reverse that of China, the United States achieves a balance in its export and import of RE trade. In addition, as is shown by Table 5, compared with other counties, the United States has a higher number of trade partners, and the number is relatively stable, indicating that its RE trade partners are quite diversified and it does not rely on just one country in both export and import. This approach can strongly protect the safety of the RE resource in the United States while strengthening its influence in the RE trade market.

**Table 5:** USA’s RE trade situation.

	Ranking index			Trade Value		Trade Volume		Number of trading partners		
	Total	Import	Export	Import	Export	Import	Export	Total	Import	Export
2002	96	98	96	10253433	6084628	1211188	1092657	27	10	25
2003	96	98	98	5315982	4109194	736764	995898	25	9	23
2004	94	96	94	3662508	6223236	670386	899446	23	9	22
2005	96	98	94	5086501	5259230	733237	531364	25	7	23
2006	96	96	96	6186832	7265717	703609	621597	24	10	21
2007	94	92	96	6632248	20934400	653460	1235699	28	6	26
2008	98	82	98	5299372	19522886	565656	1191114	33	6	33
2009	94	98	92	4976530	16544781	188136	4125516	32	7	30
2010	96	98	94	15886054	31390895	456534	1178731	31	10	30
2011	96	98	96	70579270	109027784	385968	2822054	39	9	35
2012	94	98	94	21936459	47205734	189738	1822377	31	10	30
2013	90	96	86	9624133	10707407	327785	918934	31	10	28
2014	90	96	92	5602535	3674003	290061	120952	30	10	29

(4) Germany: Germany is a country without RE resources. From the ranking index of the recent 15 years, we can see that the influence of Germany on RE trade reached the peak in 2008, subsequently, the index showed a downward trend. It can be seen from the comparison of its export figures, import figures and Table 6 that the influence of import is larger than that of export, mainly because RE import has more trade value and trade volume than RE export. Germany takes advantage of the number of trade partners, having surpassed countries with higher average ranking indices, including China, Japan, and the United States. Having more trade partners helps Germany to enhance its influence in international RE trade; in addition, having multiple means to obtain RE ensures the safety of German industries related to RE.

**Table 6:** Germany’s RE trade situation.

	Ranking index			Trade Value		Trade Volume		Number of trading partners		
	Total	Import	Export	Import	Export	Import	Export	Total	Import	Export
2002	90	90	86	1966000	926000	232144	8814	26	10	22
2003	94	96	84	3496000	170000	366594	14395	24	11	19
2004	92	94	90	3604000	94000	285713	8526	22	13	16
2005	94	96	92	4417000	82000	406760	900	23	13	17
2006	92	94	86	5568000	80000	370900	1600	25	17	17
2007	92	94	90	7744000	192000	410236	22800	23	13	16
2008	96	100	96	8783000	115000	438895	10350	21	11	16
2009	88	92	82	2909000	152000	107714	23548	25	13	22
2010	92	96	78	11384527	810910	466297	27546	32	14	23
2011	88	94	84	24408750	2491739	262278	29552	35	16	32
2012	90	96	82	17095994	936457	290080	10413	43	19	38
2013	80	90	80	5042066	510415	264013	10480	40	11	39
2014	84	92	86	4408623	421716	272181	11945	46	14	44

(5) Vietnam: Vietnam, a RE resource country, has over one million tons of reserves. According to Table 7, Vietnam has become a focus since 2008, when its ranking index increased from 0 to 96; and the index of export, 0 to 98. It can be seen from the comparison of its export figures and import figures that export has more trade value and trade volume than import does.

**Table 7:** Viet Nam’s RE trade situation.

	Ranking index			Trade Value		Trade Volume		Number of trading partners		
	Total	Import	Export	Import	Export	Import	Export	Total	Import	Export
2002	0	-	0	0	0	0	0	0	0	0
2003	2	6	0	9043	0	843	0	2	2	0
2004	10	12	0	17872	0	NA	0	3	3	0
2005	0	-	0	15750	0	NA	0	1	1	0
2006	0	8	0	10628	0	NA	0	1	1	0
2007	26	36	0	63104	0	NA	0	4	4	0
2008	0	0	0	22000	0	865	0	1	1	0
2009	96	0	98	0	12696639	0	334360	1	0	1
2010	94	52	98	3315288	19828657	49678	564708	3	1	2
2011	94	22	98	1622008	97441741	77731	1075750	4	2	2
2012	96	80	98	39332	129127191	1796	1463213	6	2	6
2013	96	8	98	1821421	84036043	127751	1961643	5	3	3
2014	98	0	100	5472448	140020174	435322	2633872	7	4	3

A policy implemented by the Chinese government reduced the RE export quotas of China by 39% in 2008 and by the same percentage in 2010 that caused a gap between supply and demand in the global RE market. The great effort that Vietnam made on exporting in 2009 has efficiently filled in the gap due to China’s policy on RE export quotas.

#### Challengers and followers analysis

In this part, we will analyze the groups of challengers and followers according to Fig 10. The two groups contain not only non-resource countries and regions (including Austria, UK, France, Norway, Hong Kong, Belgium, and Korea) but also resource countries (including India, Russia, Canada, Malaysia, South Africa, and Australia).

According to the ranking of influence, the average ranking index of non-resource countries is higher than that of resource ones. The main reason for this difference is that most of the non-resource countries shown in the figure are developed countries, who have achieved better development in industries related to RE compared to those resource countries; as a result, more demand is required by the former than the latter. The urgent demand and the lacking in RE resources of non-resource countries help them have adequate influence in the international trade market to ensure the safety of their RE industry.

Through the comparison in Table 8 below, we can see that both the number of trade partners and the trade volume of most non-resource countries are higher than those of the resource countries in 2014. Non-resource countries are much more active, especially in export trade, because of the safekeeping of the RE resource in the resource countries due to the concerns regarding environmental protection.

**Table 8:** Group of challengers and followers in the RE trade situation in 2014.

Countries	Direction	Partner	Value	Countries	Direction	Partner	Value
Austria	Export	NA	7196642	Korea	Export	6	497534
	Import	NA	4863501		Import	11	3287826
UK	Export	34	1376078	India	Export	2	341
	Import	13	5597578		Import	7	3724533
France	Export	9	202529	Russian	Export	6	498995
	Import	10	1654055		Import	5	1732732
Norway	Export	1	795	Canada	Export	4	758189
	Import	6	2726186		Import	8	2395485
HK	Export	21	21663678	Malaysia	Export	0	0
	Import	7	568291		Import	4	82374384
Belgium	Export	40	3969024	South Africa	Export	3	34733
	Import	7	95287		Import	7	23910

## Conclusion and Recommendations

In this paper, complex network theory was used to analyze the evolutionary characteristics of the international RE trade pattern. Our results can be applied to understand the trade pattern and its influencing factors of international RE trade, and furthermore, provide the basis of trade policies formulation and implementation for RE importing and exporting countries. Moreover, our model can also be used in other fields which have network structures. The model in this study is suitable to solve the problems which have the following characteristics: structural complexity, network evolution, connection diversity, dynamical complexity, node diversity, and meta-complication (Strogatz, 2001). Particularly, based on the three contributions of this paper, our model can be adopted to understand the evolution features and stability of the network and identify the key nodes in the network. For example, our model can be used to obtain the distribution structure of different species and its evolution trend and the robustness and fragility to some factors such as species richness in the studies on ecological network.

Some meaningful conclusions are drawn as follows.

First, the changing number of global RE trade communities presents a bell-like curve in the past 13 years, which is related to the policies of limitation of the production and export in China since 2002.

Second, analyzed from a globalization point of view, the relations between countries in international RE trade, become closer and the divisions among the communities become less distinct. At the same time, the actual relations between countries are not as close as indicated by their trade relations.

Third, according to the map of trade communities, their formation is largely affected by regional aspects. Most countries, except China, tend to associate with geographically closer countries to form a trade community, whereas China chooses to cooperate with geographically farther countries.

Fourth, the network of global RE trade market has a weak stability for three reasons. First, some countries do not participate in global RE trade annually. Second, the safekeeping of RE in resource countries has resulted in the condition of less supply than demand during the past 13 years; thus, countries without resources have been constantly seeking RE resources among the trade communities. Third, RE is an important resource with uneven distribution and yield, and its trade relations are built on the basis of whether a partner country has export resources. If not, then the trade relations will easily be broken.

Fifth, Japan, China, the United States, and Germany have had the most influence on global RE trade in the past 13 years. How much influence they have is positively correlated with both their trade volume and the number of their trade partners.

Based on the study, the following three suggestions are provided:

First, China, Japan, the United States, and Germany should cooperate in the international RE trade market to maintain its stability. According to its network, the number of countries in the trade communities related to these four countries account for over 80% of the total amount. The dispute that occurred in 2012 between the communities of the United States, Japan, and Europe and China in the WTO led to the stability decreasing to its lowest point in history. Thus, those four countries should work together to avoid such situation.

Second, RE resource countries, except China should be more responsible in the global RE export market to ensure its stability, the diversity of RE source and the safety of the global RE industry. According to the import figures and export figures of major RE trade countries in 2014 in Table 8, there is a huge gap between trade countries in the groups of “Leaders”, “Challengers,” and “Potential leader” and resource countries, except China and Vietnam, who are not performing well enough in both the number of export partners and export figures. With the continuous development of industries related to RE, the demand of RE continues to grow. Trade countries should fulfill their common responsibility for global export market to guarantee a sound development of RE industry.

Third, trade-participating countries, especially resource ones, can enhance their influence on international market by raising the trade volume or the number of trade partners. According to the analysis above, how much influence a country has is positively correlated with both of its trade volume and the number of its trade partners. Thus, resource countries can enhance their influence by raising both of the two factors above; as for countries without RE resource, they can strengthen their influence by increasing the number of trade partners if import figures cannot be guaranteed.
